# The Use of Vesecants in Diseases of Children

**Published:** 1876-06

**Authors:** J. M. Lewis

**Affiliations:** Kosciusko, Mississippi


### THE USE OF VESECANTS IN DISEASES OF CHILDREN.

By J. M. LEWIS, M.D., Kosciusko, Mississippi.

Where do physicians get their authority for the use of large blisters, in treating diseases of children? Certainly not from any late works on diseases of the same. Prof. Vigel, in his work, (Diseases of Children,) does not mention their use in the treatment of pneumonia, (the disease more than all others where blisters are used,) but uses tepid compresses as a local application. Dr. J. Lewis Smith, in his excellent work, (Diseases of Children,) recommends counter-irritation, but he does not recommend vesecating a large surface. He says, (speaking of the treatment of pneumonia,) "when the inflamation is extreme, and the symptoms urgent," blister with cantharidal collodion, a half dozen spots, more or less, as large as a ten cent piece.

Drs. Meigs and Pepper, in their large and valuable work, (Diseases of Children,) say, (in giving their treatment of pneumonia,) "We rarely employ blisters, but prefer mustard poultices. Dr. West does not employ blisters in the treatment of the same disease, and Rilleth and Barthez were of the opinion that blisters did not exert the least influence upon any one of the symptoms of pneumonia, but that, on the contrary, they increased the fever. It may be that I have not access to the best authorities on the subject, but my own judgment and experience teaches me that it is pernicious. I have often seen, in consultation, children, when in my opinion and the word of the child, the principal suffering was from the blistered surface.

It may be that I am on the other extreme, but I have never applied a blister to a child yet, and have seen no reason to regret it. While I often employ counter irritation, I do not (without an accident) blister to obtain it. Physicians, sometimes, seem to forget what a delicate structure the cuticle of a child is, and that in many cases it prolongs convalesence, besides causing unnecessary pain. It is useless for any one to say that large blisters are not used to any great extent, for they are, and by men who, in many instances, enjoy a good practice.

It is to be hoped that physicians will be more cautious in the use of vesecants with children, for however much benefit may be claimed for them in the strong and well-developed adult, their use is certainly questionable in the young and aged.

In this short paper, I have not spoken of the supposed benefits, results, or depressing effects of blisters, my object being only to call attention to the too frequent use in diseases of children.

